# Be prepared for interruptions: EEG correlates of anticipation when dealing with task interruptions and the role of aging

**DOI:** 10.1038/s41598-024-56400-y

**Published:** 2024-03-07

**Authors:** Soner Ülkü, Stephan Getzmann, Edmund Wascher, Daniel Schneider

**Affiliations:** Leibniz Research Centre for Working Environment and Human Factors (IfADo), Ardeystraße 67, 44139 Dortmund, Germany

**Keywords:** Task interruptions, Working memory, Selective attention, EEG, Aging, Anticipation, Human behaviour, Psychology, Cognitive ageing, Attention, Cognitive control, Neuroscience, Short-term memory, Working memory

## Abstract

Dealing with task interruptions requires the flexible use of working memory and attentional control mechanisms, which are prone to age-related changes. We investigated effects of age on dealing with task interruptions and potential advantages of anticipating an interruption using EEG and a retrospective cueing (retro-cue) paradigm. Thirty-two young (18–30 years) and 28 older (55–70 years) participants performed a visual working memory task, where they had to report the orientation of a target following a retro-cue. Within blocks of 10 trials, they were always, never, or randomly interrupted with an arithmetic task before the onset of the retro-cue. The interruption-induced decline in primary task performance was more pronounced in older participants, while only these benefited from anticipation. The EEG analysis revealed reduced theta and alpha/beta response to the retro-cue following interruptions, especially for the older participants. In both groups, anticipated interruptions were associated with increased theta and alpha/beta power prior and during the interruption, and stronger beta suppression to the retro-cue. The results indicate that interruptions impede the refocusing of attention on the task-relevant representation of the primary task, especially in older people, while anticipation facilitates preparation for the interruption task and resumption of the primary task.

## Introduction

With the widespread use of technology and the constant need for multitasking, task interruptions have become an inherent part of our daily lives^1^. How well we can cope with these interruptions depends largely on the extent to which working memory and attentional control processes ensure the transition between the processing of the primary and interrupting tasks. These processes are prone to non-pathological cognitive aging^2^, which may be a reason for the frequently reported age-related difficulties in coping with interruptions^3–6^. The current study investigated these relationships using neurophysiological measures, and explored to what extent the possibility to anticipate an upcoming interruption can act as a potential factor to compensate for age-related deficits.

Several cognitive processes are involved in processing a task interruption, which all may be affected by age-related changes. These changes include (but are not limited to) working memory, episodic memory, information processing speed as well as cognitive control^5–7^. However, to relate these general changes to age-related deficits in processing interruptions, it is necessary to identify which cognitive processes are relevant here: When a shift has to be made from a currently pursued primary task to an interrupting secondary task, it is necessary to temporarily store the current processing state of the primary task and all relevant information that will be required later for resuming this task. Then attention has to be directed towards the processing of the interruption. After completion of the interrupting task, attention must be re-directed to the primary task, leading to a reactivation of task-relevant information in working memory. This last step might also involve the inhibition of the now irrelevant information from the interrupting task.

The phenomenon of shifting the focus of attention between tasks can be studied using retrospective cuing (retro-cue) paradigms. These paradigms involve the storage of information about future tasks in working memory and make use of cues presented during the storage phase to direct attention to specific content^8,9^. While it is not entirely clear whether such attentional control processes at the level of working memory are impaired^10,11^ or unimpaired in higher age^9,12,13^, there is evidence that age-related deficits are especially apparent when working memory storage is interrupted by a cognitively demanding task^14,15^.

One possible approach to help counteracting such age-related deficits might be to provide opportunities to prepare for an interruption. In task-switching, for example, numerous studies have revealed that the time it takes to switch between tasks can be significantly decreased if participants are given a preparatory period before the new task begins^16,17^. Likewise, having knowledge about an upcoming interruption can mitigate its negative impact on the performance of the primary task^18–20^. It is important to note in this regard, that the processing of interruptions is intrinsically linked to the function of task switching. Both are based on shifting attention between two tasks and thus rely on attentional control processes and working memory^21^. More time for this process should make it possible to better disengage attention from the no longer relevant information in working memory and shift the focus of attention to the relevant task^18–20^.

The extent to which the preparation for an interruption might differ between younger and older individuals was investigated in a study by Arnau and colleagues^3^. Participants performed a working memory task that was randomly interrupted by a cued math task. Interestingly, only younger adults showed neurocognitive correlates of preparatory (or proactive) increases in attentional control, as indexed by increased power of frontal midline theta oscillations in response to the cues signaling an upcoming interruption. No such preparatory effect was observed in older adults. This suggests that at least on a trial-by-trial basis, with cues presented only shortly before the interrupting task (here: 500 ms), cognitive preparation is not effective in reducing age-related deficits in processing interruptions. It is further possible that due to reduced cognitive resources and a generally lower speed of information processing^22^, the older adults were simply not able to efficiently use the cues in the fast sequence of stimuli. They might rather benefit from being able to anticipate the processing of an interruption over a longer period. In the current study, we used an interrupted working memory task including a retro-cue after the interruption phase. This design allows for investigating the extent to which attentional control processes engaged after presentation of the retro-cue get impaired by a prior task interruption^15^. Importantly, we further implemented a blocked design allowing participants to anticipate the upcoming trial structure, thereby providing sufficient time for cognitive preparation (for a more detailed explanation of the paradigm please refer to the “Methods” section).

For a high-resolution temporal mapping of the neural correlates of the processing of the interruption and the reorienting of attention to the primary task, neural oscillations were measured using EEG. Previous studies could show that the orienting of attention towards specific content in working memory is reflected in changes in the power of delta to theta (~ 2–7 Hz) and alpha to beta (~ 8–30 Hz) oscillations. For example, oscillatory power in the alpha (~ 8–14 Hz) and beta frequency range (~ 15–30 Hz) was shown to be suppressed at posterior recording sites when working memory content was cognitively manipulated^23^, and when attention was directed to a subset of information stored in working memory^24^. Oscillatory power in the delta/theta frequency range was found to be increased when the focus of attention had to be switched between working memory representations of two different tasks^25^. The strength of this effect was correlated to the extent of hemispheric asymmetries of posterior alpha power reflecting the spatial orienting of the focus of attention following a retro-cue. In line with earlier research linking the fronto-central oscillatory response in the theta frequency range to attentional control processes^26–29^, this supports the notion that delta/theta power modulations reflected a top-down control process required for shifting attention on the level of working memory^25^. Importantly, these oscillatory effects were found to be modulated by a prior interruption. When participants had to select one out of two working memory representations for later report, the increase in theta power and the suppression of posterior alpha power following the retro-cue was reduced when an interrupting task preceded it, compared to a phase without interruption. This suggested a deficit in retrospective attentional processes following a task interruption^15,30^.

Drawing from these previous findings, we hypothesize the presence of age-related effects concerning the capacity to engage in a working memory task when having to face an interruption. We further expect to observe age-related variations in how individuals employ the anticipation of a task interruption to compensate for resulting performance decrements. More specifically, it is first expected that an interruption decreases performance in the primary working memory task in older adults more than in younger adults. Secondly, the decrease in performance in the primary task should be less pronounced when the interruption can be anticipated. In other words, the interruption-related decrease in performance should be smaller in interruption blocks (in which the interruption could be anticipated) than in random blocks (in which the interruption could not be anticipated). Finally, assuming that older adults use the anticipation of the experimental conditions (interruption vs. random blocks) as a strategy for compensation, this anticipatory benefit should be even stronger in the older group. On EEG level, we expect to replicate previous findings of reduced frontal theta and less suppression of posterior alpha/beta power during retrospective attentional orienting when a task interruption preceded retro-cue presentation^30^. Moreover, these oscillatory effects should be further modulated by the opportunity to anticipate the presence or absence of a task interruption, with neural oscillatory signatures of retrospective attentional processes being stronger when anticipation was possible. If especially older individuals make use of the possibility to anticipate the experimental conditions, the effects on frontal theta and posterior alpha/beta oscillations should be stronger for this group. This would also be in line with the notion that older adults employ strategies to cope with age-related cognitive decline, such as making use of compensatory neural activity or recruiting larger neural circuits to perform a cognitive task at a level similar to younger participants^31,32^. These hypothesized result patterns would enable a better interpretation of the neurocognitive processes necessary for processing an interruption during a working memory task. Furthermore, conclusions could be drawn about possible compensatory mechanisms for age-related deficits in the processing of interruptions, which would also allow more precise recommendations for the everyday handling of this kind of interference.

## Results

### Behavioral data

**Figure 1:**
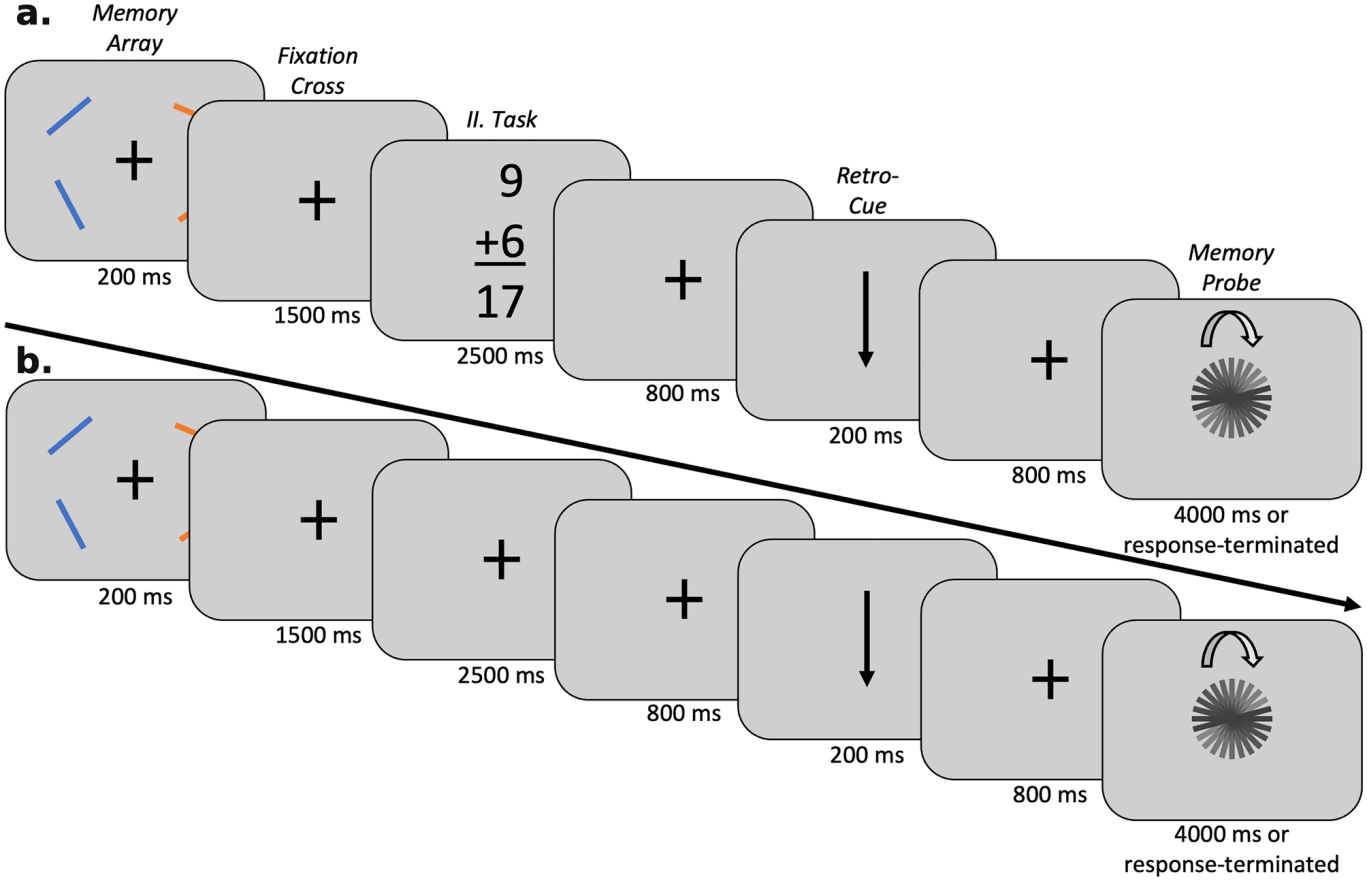
Structures of trials with interruptions (**A**) and without interruptions (**B**). A. Structure of an interruption trial where the participants had to attend an interrupting task in-between the presentation of the memory array and the retro-cue. All participants were assigned a target color at the beginning of the experiment and were instructed to store only the orientation of the bars presented in this color (orange vs. blue). After the participant was presented with the memory array, there was a delay of 1500 ms with a fixation cross on the screen before they were presented with the secondary task for a duration of 2500 ms. After this, there was an additional fixation period of 800 ms before they were presented with the retro-cue indicating which bar’s orientation to report. (**B**) Structure of a no-interruption trial where the fixation interval between the memory array and the retro-cue was prolonged for 2500 ms.

**Figure 2:**
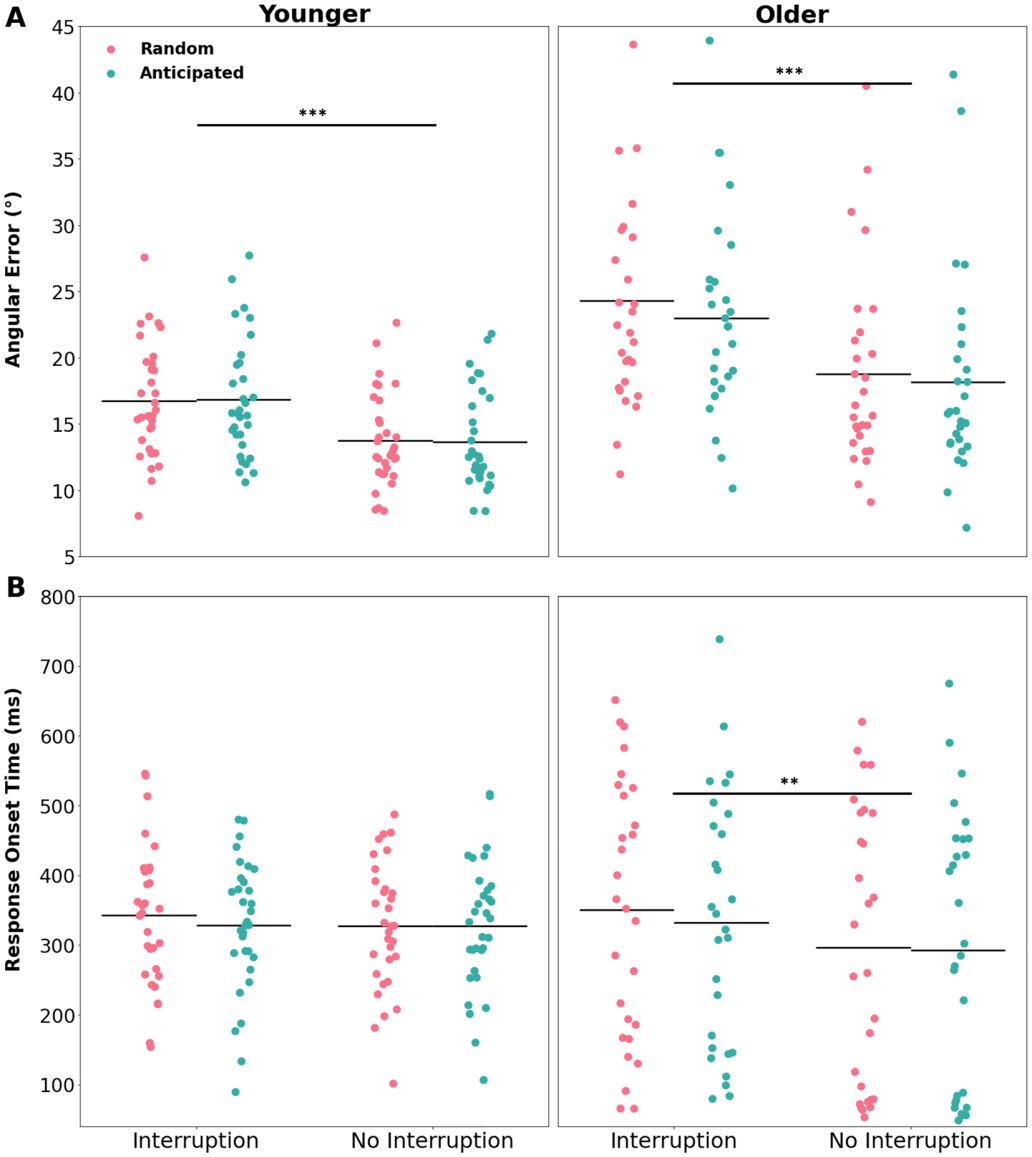
Behavioral parameters from the primary task. (**A**) Angular error and (**B**) response onset time of younger and older participants are shown separately for interruption and no-interruption trials, and for anticipated and random conditions. The colored dots represent the individual values, and the horizontal lines indicate mean values for the given parameter. The asterisks represent the significant differences between experimental conditions (*p* < .001 ***, *p* < .01 **, *p* < .05 *).

The current experiment was based on a visual working memory task requiring the report of the orientation of a remembered object (a randomly oriented bar) and an interrupting arithmetic task (see “Methods” section, Fig. 1). The orientation report for the cued bar was accomplished by horizontal movement of the computer mouse with the right hand. Therefore, task accuracy in the primary task was measured as the difference in degree between the original orientation of the cued bar, as presented in the memory array, and the orientation adjusted upon presentation of the memory probe. As an equivalent of response times, we further measured the time required to first move the computer mouse after presentation of the memory probe (see Fig. 2).

As expected, performance in the working memory task was compromised by the interrupting task (see Fig. 2A, accuracy: F(1,55) = 157.85, *p* < 0.001, *η*_*p*_^*2*^ = 0.74; Fig. 2B, response onset times: F(1,55) = 13.00, *p* < 0.001, *η*_*p*_^*2*^ = 0.19). Furthermore, based on the previously formulated hypotheses, we analyzed whether older adults featured a stronger impairment of primary task performance after interruptions. This was supported by a significant age*interruption interaction which was evident both for task accuracy, *F*(1, 55) = 11.31, *p* < 0.001, *η*_*p*_^*2*^ = 0.17, and for response onset times, *F*(1, 55) = 6.12, *p* = 0.02, *η*_*p*_^*2*^ = 0.10 (Fig. 2A,B). Additionally, while we expected a reduced effect of the interruption on primary task performance when participants could anticipate the interruption condition (compared to the random blocks), this effect was neither evident for task accuracy, *F*(1, 55) = 0.07, *p* = 0.91, *η*_*p*_^*2*^ = 0.00, nor for response onset times, *F*(1, 55) = 3.60, *p* = 0.06, *η*_*p*_^*2*^ = 0.06. We further hypothesized that the possibility to anticipate the absence vs. presence of an interruption would be differently used in the younger vs. older group. This was supported by an age*anticipation interaction for task accuracy, *F*(1, 55) = 5.17, *p* = 0.03, *η*_*p*_^*2*^ = 0.09 (but not for response onset times: *F*(1, 55) = 1.08, *p* = 0.3, *η*_*p*_^*2*^ = 0.02), which was independent of the actual presence vs. absence of an interruption, *F*(1, 55) = 0.40, *p* = 0.53, *η*_*p*_^*2*^ = 0.01. As expected, older adults were more accurate in the primary task when they could anticipate the interruption (anticipated vs random: *t*(24) = −2.64, *p*_*corr*_ = 0.03, *cohen’s d* = 0.19, 95% CI [−1.86, −0.23]). This effect was not evident in the younger group (anticipated vs random: *t*(31) = −0.05, *p*_*corr*_ = 0.96, *cohen’s d* = 0.0, 95% CI [−0.54, 0.51]).

**Figure 3:**
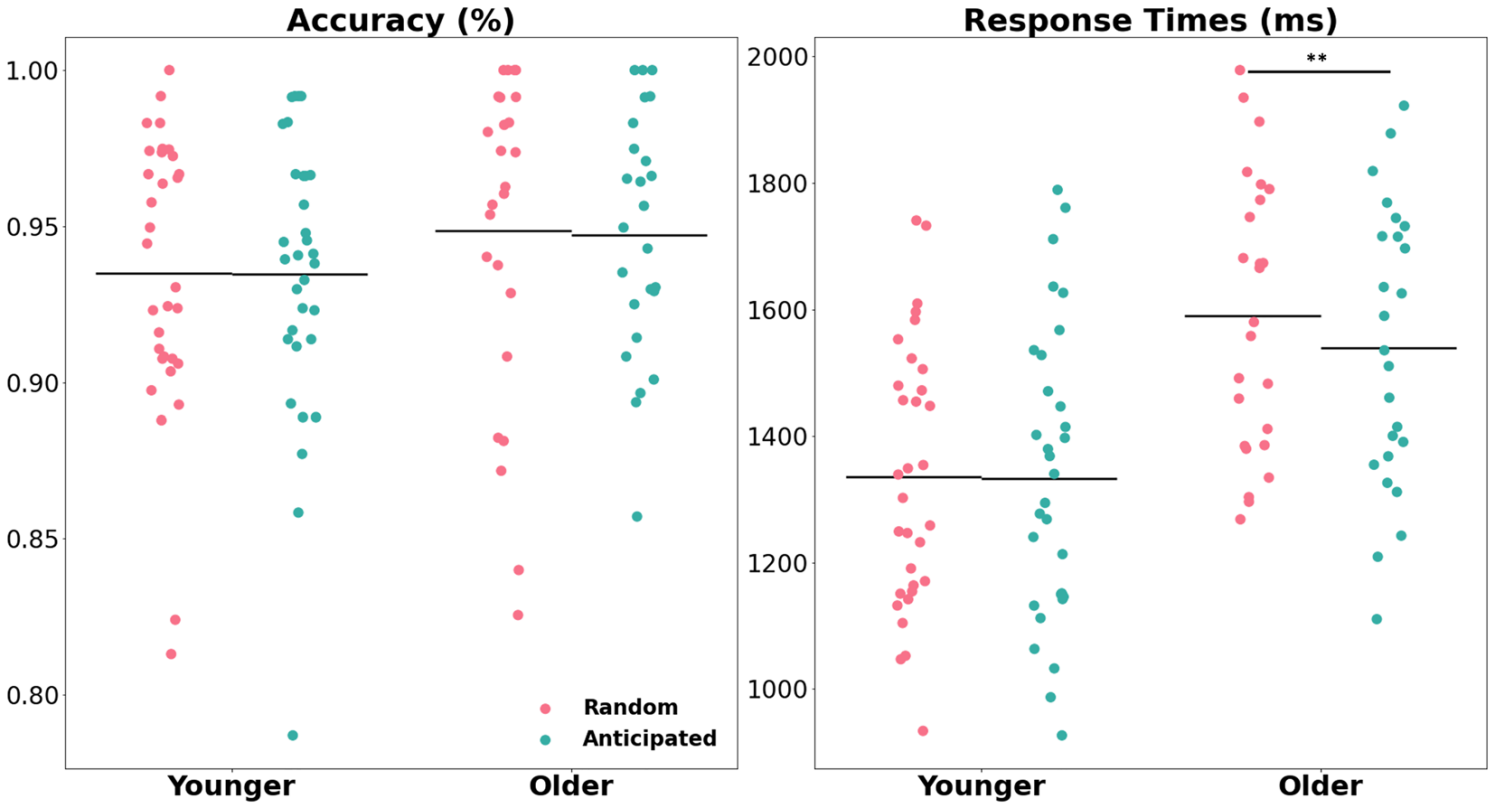
Accuracy and response times for the interrupting task. The results are presented separately for each age group and for the anticipated vs. random conditions. The colored dots represent the individual values and the horizontal lines indicate mean values for the given parameter. The asterisks represent the significant differences between experimental conditions (*p* < .001 ***, *p* < .01 **, *p* < .05 *).

The analysis of the behavioral parameters obtained from the interrupting task did not indicate differences in accuracy between anticipated and random interruptions (anticipated: *M* = 94%, *SD* = 6%; random: *M* = 93%, *SD* = 7%), *F*(1, 56) = 0.01, *p* = 0.94, *η*_*p*_^*2*^ = 0.00, nor between age groups (young: *M* = 0.93, *SD* = 0.04; old: *M* = 0.94, *SD* = 0.08), *F*(1, 56) = 0.00, *p* = 0.98, *η*_*p*_^*2*^ = 0.00 (no age*anticipation interaction, *F*(1, 56) = 0.02, *p* = 0.89, *η*_*p*_^*2*^ = 0.00). However, response times in the interruption task were faster when the interruption was anticipated (anticipated: *M* = 1407.32 ms, *SD* = 273.30; random: *M* = 1428.40 ms, *SD* = 281.87), *F*(1, 56) = 7.56, *p* = 0.01, *η*_*p*_^*2*^ = 0.12. This effect further differed as a function of age (age*anticipation interaction, *F*(1, 56) = 7.60, *p* = 0.01, *η*_*p*_^*2*^ = 0.12; see Fig. 3). Post-hoc t-tests revealed that the anticipation effect was only evident for the older participants (anticipated vs. random: *t*(25) = −3.49, *p*_*corr*_ < 0.01, *cohen’s d* = 0.14, 95% CI [−70.81, −18.25]), while no difference occurred for younger participants (anticipated vs. random: *t*(31) = −0.22, *p*_*corr*_ = 0.83, *cohen’s d* = 0.01, 95% CI [−20.93, 16.87]).

### Electrophysiological data

**Figure 4:**
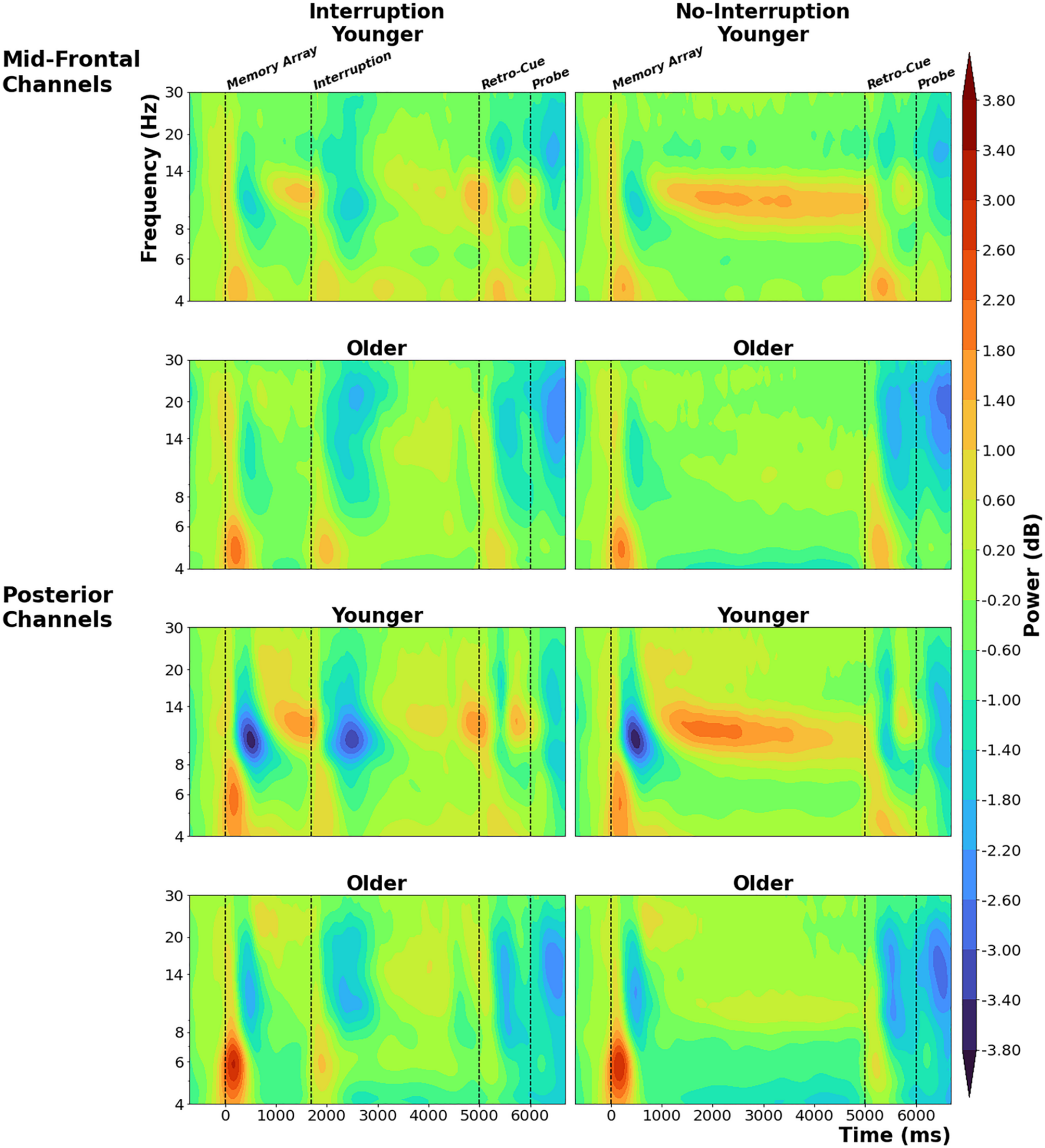
Time–frequency plots per age group and interruption condition (averaged across anticipation conditions). The values on the spectra are baseline-corrected oscillatory power measures. The upper four subplots are from a cluster of mid-frontal channels (Fz, F1, F2, FC1, FC2) and the lower four subplots are from a posterior channel cluster (PO3, POz, PO4, O1, Oz, O2). The events (onsets of memory array, interruption, retro-cue, and probe) are marked by vertical dotted lines.

To provide a descriptive overview of the oscillatory responses over the whole trial, we first present the event-related spectral perturbations from mid-frontal and posterior channels per interruption condition and age group (Fig. 4). In addition to the increase in theta power (~ 4–7 Hz) and the suppression of alpha and beta power (~ 8–30 Hz) after presentation of the respective stimuli (memory array at 0 ms; interruption or prolonged fixation phase at 1700 ms; retro-cue at 5000 ms; memory probe at 6000 ms), younger subjects in particular showed a sustained increase in alpha power (~ 10 Hz) during the working memory delay phase. This effect is superimposed by the stimulus-evoked theta power increase and the suppression of alpha/beta power in the conditions with interruptions.

These time–frequency decompositions were used to run cluster-based permutation statistics. This was first done on the whole sample (i.e., without differentiating between age groups) by permuting either the anticipation or interruption conditions (see within-subject analyses). Following this step, the time–frequency decompositions were further analyzed using age as a factor. This was done by running the cluster-based permutation statistics on anticipation and interruptions effects, e.g., running the tests by calculating the time–frequency differences for random minus anticipated interruptions, and comparing the age groups for these measures. More detailed information about the exact parameters and statistical tests can be found in the “Methods” section. In case the different clusters were largely overlapping across frequency bands (such as the cluster observed across both alpha and beta bands), we refer to multiple frequency bands in the naming of the clusters.

### Within-subject analyses

**Figure 5:**
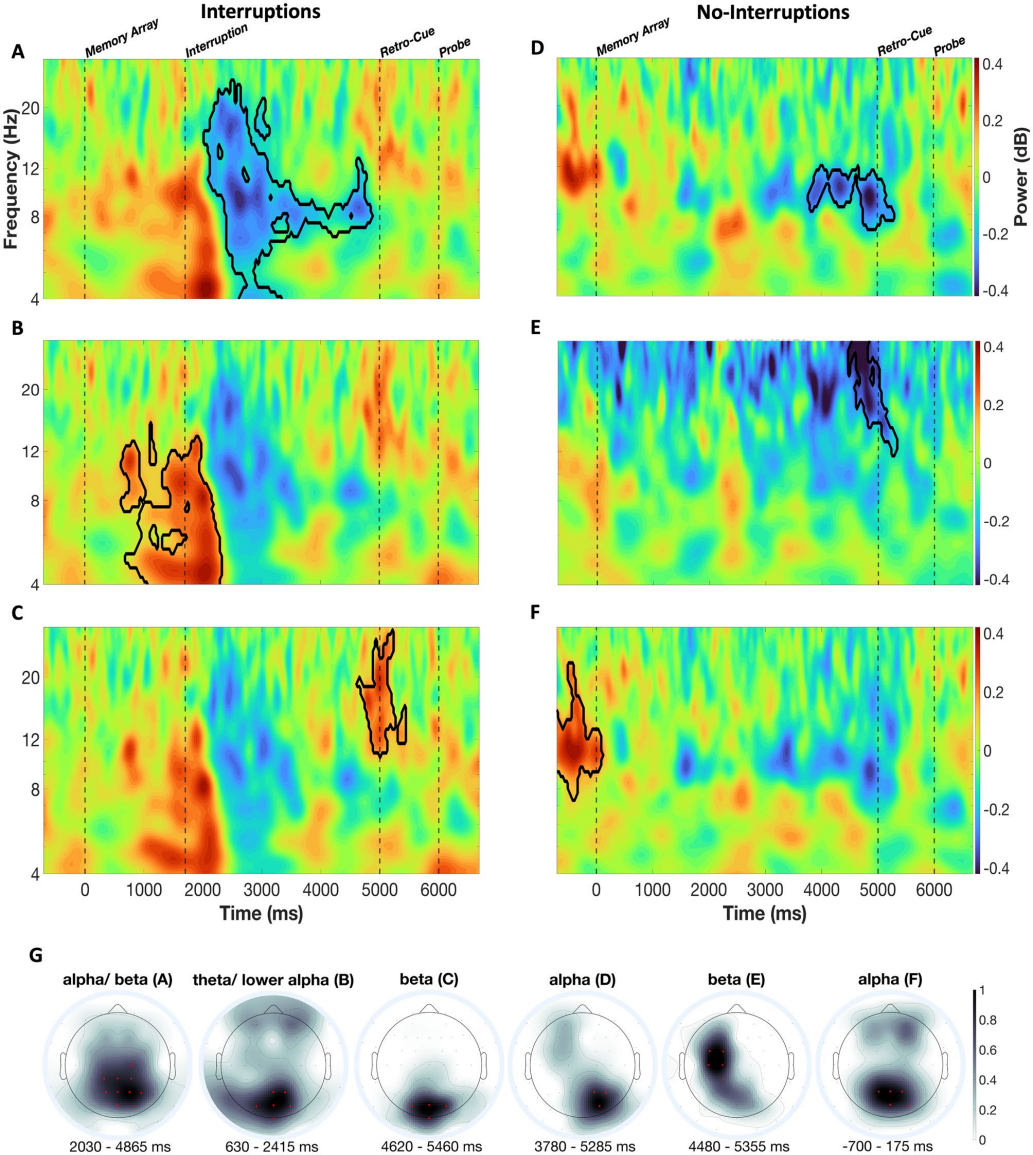
Time–frequency plots for the effects of anticipation independent from age. The left three subplots (**A**, **B**, and **C**) show the difference between random and anticipated interruptions (i.e., random minus anticipated), whereas the right three subplots (**D**, **E**, and **F**) show the difference between random and anticipated conditions without interruption. The significant clusters are marked with black outline for these comparisons. The events (onsets of memory array, interruption, retro-cue, and probe) are marked by vertical dotted lines. The topographies (**G**) are shown for each cluster per comparison, indicating how strongly each channel contributed to the overall cluster (see “Methods” section). Channels with values equal to or higher than 0.7 are marked in red. These channels were also used for the time–frequency plots (**A**–**F**).

The cluster-based permutation test on the effects of anticipation revealed six clusters, in which the power measures differed between random and anticipated interruptions (see Fig. 5A–C), and between random and anticipated no-interruptions (see Fig. 5D–F). The first cluster (*t-*sum = −48,242.97, critical *t* = −5325.57, *p* = 0.001) is centered around the alpha and beta frequency bands. It has a posterior (parietal) topography and occurred around the time when the participants processed the interrupting task (Fig. 5A), indicating that during randomly occurring interruptions participants exhibited lower oscillatory power in alpha and beta frequency range than during anticipated interruptions. The second cluster (*t-*sum = 48,360.66, critical *t* = 5206.03, *p* = 0.001) features strongest effects at parieto-occipital electrodes and occurred before and after the onset of the interrupting task. The cluster indicates that higher power of theta oscillations was shown for randomly occurring interruptions (Fig. 5B). Channels with significant time–frequency points of this cluster seemed to be additionally localized over frontal brain areas (without exceeding the 0.7 threshold). The last cluster (*t-*sum = 5243.33, critical *t* = 5206.03, *p* = 0.05) in this comparison is centered in the beta frequency band around the onset of the retro-cue. It indicates that there was stronger suppression of oscillatory power following an anticipated task interruption (Fig. 5C).

The first cluster (*t-*sum = −6012.4, critical *t* = −5508.7, *p* = 0.04) from the comparison of random and anticipated no-interruption trials is localized around right posterior channels and in the alpha frequency band, starting before the presentation of the retro-cue. Participants exhibited more oscillatory alpha suppression following random no-interruptions, in contrast to anticipated no-interruptions (Fig. 5D). The second cluster from this comparison (*t-*sum = −7851.5, critical *t* = −5508.7, *p* = 0.02) was localized above the left-hemispheric motor cortex, with the time–frequency points being centered around the beta frequency band and starting slightly before the presentation of the retro-cue. It indicates that there was a stronger suppression of beta power within the random condition than within the anticipated condition (Fig. 5E). The last cluster (*t-*sum = 11,846, critical *t* = 5775.7, *p* = 0.01) was localized around the posterior channels, with the significant time–frequency points being centered around the alpha frequency band, immediately before the trial began. It indicates that participants exhibited lower oscillatory alpha power in anticipated, than random, no-interruption trials even before the initial stimulus was presented (Fig. 5F).

We further contrasted the interruption and no-interruption conditions by means of the cluster-based permutation statistical approach, averaged across anticipated and random conditions. Since the secondary task induced strong oscillatory responses during the interruption period, only the time window following the retro-cue (i.e., 5000 ms after trial onset) was considered for this analysis. There were two significant clusters showing that the participants exhibited higher oscillatory power in the theta band (*t-*sum = −48,806, critical *t* = −10,063, *p* = 0.001) and lower oscillatory power in the alpha/beta frequency band (*t-*sum = 153,800, critical *t* = 8933, *p* = 0.001) in no-interruption trials than in interruption trials. These results are visualized in supplementary Fig. S1.

### Age-dependent analyses

**Figure 6:**
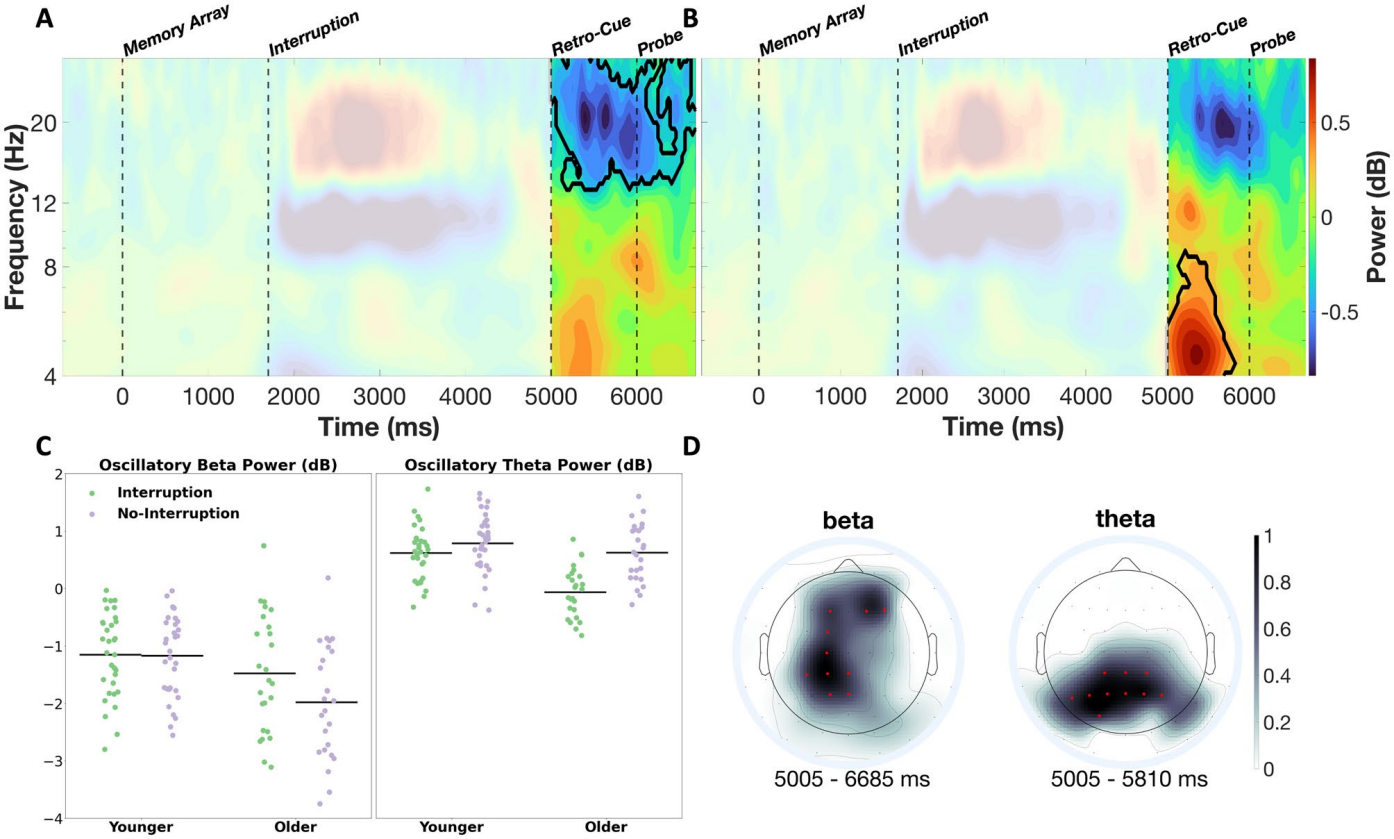
Time–frequency plots for the interaction of interruption condition and age. The upper two subplots (**A** and **B**) show the difference between age groups (younger minus older participants) that is calculated on already subtracted interruption vs. no-interruption conditions. The significant clusters are marked with black outline for these comparisons. The events (memory array, interruption, retro-cue, and probe) are marked by vertical dotted lines. Scatterplots (**C**) show the averaged power values for each cluster from the time–frequency plot per participant and condition, as well as the respective mean values. The topographies (**D**) reflect the contribution of each channel to the overall cluster (see “Methods” section). Channels with values equal to or higher than 0.7 are marked in red. These channels were also used for the time–frequency plots (**A** and **B**).

Following the analyses of main effects on neural oscillatory patterns, the interaction between age and either of the two within-subject factors was statistically tested (see methods section for further details). There were no significant clusters found for random vs. anticipated interruptions, or random vs. anticipated no-interruptions. However, two significant clusters were found for age effects on the difference between interruption and no-interruption trials (see Fig. 6). The first cluster (*t-*sum = −30,348, critical *t* = −7918.4, *p* < 0.01) was found in the higher-alpha and beta frequencies right after the retro-cue, showing that the reduced alpha/beta power suppression following interruptions (as compared to trials without interruption) was stronger for the older than for the younger participants (Fig. 6A and C). This interaction featured a left-hemispheric centro-parietal and frontal topography (Fig. 6D). The second cluster (*t-*sum = 12,894, critical *t* = 8350.6, *p* = 0.03) that stood out in this comparison was found in the theta frequency range and appeared right after presentation of the retro-cue. Participants exhibited reduced oscillatory theta response to the retro-cue following interruptions, and older participants showed a stronger effect in this regard (Fig. 6B,C).

To disentangle these interactions, post-hoc t-tests were run on the average power—gathered from these two clusters separately for age groups, and interruption and anticipation conditions. These post-hoc tests revealed that for the alpha/beta cluster the effect was actually driven by older participants (interruption vs. no-interruption: *t*(24) = 5.06, *p*_*corr*_ < 0.001, *cohen’s d* = 0.5, 95% CI [0.3, 0.71]), while no difference occurred for younger participants (interruption vs no-interruption: *t*(31) = 0.48, *p*_*corr*_ = 0.64, *cohen’s d* = 0.03, 95% CI [−0.06, 0.1]). The theta cluster showed a similar pattern, with a stronger difference between conditions for the older participants (interruption vs. no-interruption: *t*(24) = −9.3, *p*_*corr*_ < 0.001, *cohen’s d* = 1.46, 95% CI [−0.85, −0.54]) than for the younger participants (interruption vs. no-interruption: *t*(31) = −2.31, *p*_*corr*_ = 0.05, *cohen’s d* = 0.32, 95% CI [−0.29, −0.02]). The statistical analyses for a further three-way interaction between the factors age, interruption, and anticipation yielded no significant results for the alpha/beta, *F*(1, 55) = 0.01, *p* = 0.93, *η*_*p*_^*2*^ = 0.00, nor for the theta cluster, *F*(1, 55) = 2.56, *p* = 0.12, *η*_*p*_^*2*^ = 0.04.

## Discussion

In this study we investigated the attentional control mechanisms required when dealing with interruptions, potential benefits resulting from the possibility to anticipate an upcoming interruption, and how cognitive aging may influence these processes. Using a retro-cue paradigm, younger and older participants performed a visual working memory task, which was sometimes interrupted by an arithmetic task. Participants were informed whether interruptions would occur in the upcoming 10 trials. In addition to behavioral data, we measured neural oscillations via EEG as correlates of the processing of the interruption and the resumption of the primary task. In the following, we discuss the effects of anticipation on the processing of task interruptions and the role of age, with a particular focus on the extent to which older people are more affected by interruptions and might benefit more from anticipation than younger people.

In line with numerous previous studies^30,33–35^, interruptions reduced primary task performance, as indicated by higher angular errors in the working memory task when the secondary task had to be performed in-between. This effect of interruptions was more pronounced in the older group, suggesting that older participants’ working memory performance suffered more from interruptions than their younger counterparts. The interruption-induced decline in performance of the older participants was reduced however, when they were able to anticipate the interruptions (see Fig. 2). Interestingly, the positive effect of anticipation was observed irrespective of whether there actually was an interruption or not. That is, even when the primary task was not interrupted, performance was better than in trials without an interruption in the ‘interruption random’ condition. Given the block-wise design, in which the information of whether an interruption would occur or not was provided for the upcoming 10 trials, the effect of anticipation suggests that older participants efficiently use the opportunity to prepare for the upcoming experimental situation. Thus, in case of an anticipated interruption or when an interruption might occur, they might have reserved cognitive resources for the processing of the interrupting task, while in trials without interruption (i.e., ‘no interruption anticipated’ condition) they might have fully engaged all resources on the working memory task. In line with that, not only did the primary task benefit from anticipation in the older group, but also the interrupting task, as indicated by faster responses than with random interruptions. In contrast, no such anticipation benefits on performance in the working memory task or the interrupting task were observed in the younger group. A further explanation for this pattern of results could be that the mere awareness of the exact structure of the following 10 trials (i.e., an interruption will be present vs. absent) had a positive influence on task performance. Older participants in particular would benefit from such preparation, as cognitive flexibility typically decreases with age and rapid switching between task sets becomes more challenging^36^. In this case, it would not be about age-related differences in dividing cognitive resources between the primary task and the interruption, but about using additional information about the upcoming tasks to compensate for deficits.

### Effects of interruptions on EEG correlates of attentional control processes following the retro-cue

Effects of interruptions and the possibility to anticipate whether an interruption will occur have also been observed on the EEG level. First, we discuss differences in neural oscillations caused by the interrupting task. Therefore, the time windows analyzed refer exclusively to the period after the presentation of the retro-cue. The comparison of interruption and no-interruption trials averaged across the anticipation and random condition revealed a reduced theta response and a lower suppression of higher-alpha and beta power after the retro-cue when an interruption preceded it (see supplementary Fig. S1). This result is a replication of an earlier study which was based on a comparable task design^30^. Prior research indicated increases in theta power and suppression of posterior alpha/beta power when attention had to be focused on certain working memory content^24,25^. The present pattern can thus be associated with a deficit in the retrospective orienting of attention to the task-relevant working memory content following an interrupting task. At least for alpha and beta oscillations, this interpretation is further supported by the finding that strong suppression of oscillatory power after the retro-cue is associated with high performance in the working memory task (see supplementary materials for more information).

A comparison of the younger and older participants showed that theta power as a response to the retro-cue was decreased in both groups after an interruption, but significantly more so in the older group (see Fig. 6B,C). This age * interruption interaction was strongest at a broad cluster of posterior electrodes, which might be related to earlier observations that the increase of theta power at both mid-frontal and more posterior sites predicted behavioral performance in situations requiring attentional control (for example in the Stroop task:^37^). Reduced higher-alpha and beta power after interruptions, on the other hand, was only found for the older, not for the young participants (see Fig. 6A and C). Both effects correspond well with the stronger decline in performance observed in the older group and suggest that interruptions caused greater problems for older participants in resuming the primary task (also known as ‘interruption recovery failure’) 35. This pattern is consistent with previous EEG findings on age-related deficits in switching between relevant task goals^39^. Moreover, these results parallel previous findings on age-related deficits in retro-cue based orienting of attention in working memory^10,11^. Here, too, behavioral results have shown that such a deficit is primarily associated with preceding task interruptions^14^. It is also important to emphasize that the reduced suppression of oscillatory power after the retro-cue in the older group compared to the younger group was mainly evident in the higher alpha and beta frequency range and at left hemispheric central and centroparietal electrodes. Such alpha and beta power suppression can be associated with motor planning processes when it appears contralateral to the responding hand. This was the case in the current experiment, because participants always had to respond to the working memory task by moving the computer mouse with the right hand. Comparable to previous retro-cue based working memory tasks^24,40–42^, participants in the current experiment were able to plan the movement required to adjust the orientation of the memory probe from the time the retro-cue was presented, as it was clear from this point which orientation should be reported. Prior research has shown that interruptions lead to a disturbed reactivation of motor representations in working memory^43^. The current results thus suggest that this interruption-related deficit occurred primarily with the older adults.

These age-dependent deficits are also well in line with neurobiological changes in the course of non-pathological aging. For instance, it is known that age-related cognitive decline is related to changes in brain structure and function. At the structural level, aging is associated with decreased gray and white matter in the prefrontal cortex (PFC), but also in the medial temporal and parietal cortices. In addition, aging is associated with a deficient dopamine system, particularly with respect to PFC function^32,44^. These changes are particularly relevant for the ability to deal with interruptions, as the brain areas concerned are strongly associated with selective attention, inhibitory control, and working memory.

### Oscillatory effects in the EEG related to anticipation

Contrasting anticipated and random conditions revealed the potential benefit of anticipating the interruption condition. In case of an anticipated task interruption, participants showed lower theta power already slightly before and following the onset of the interruption at a broad cluster of posterior and anterior channels (see Fig. 5B). Additionally, alpha/beta power was reduced during the processing of the interrupting task (mainly at a cluster of parietal electrodes; see Fig. 5A). While increased theta power has generally been linked to the engagement or implementation of cognitive control^45–47^, the suppression of alpha power after the presentation of new sensory input signals the allocation of attention for the processing of task-relevant information^48^. With reference to the current results, this could mean, firstly, that less cognitive control for processing of the interruption needs to be engaged during the working memory storage phase if it is already clear at the beginning of each trial that an interruption will occur. The weaker suppression of alpha/beta oscillatory power after anticipated task interruptions may further indicate that the directing of attention away from the primary task and towards the processing of the interrupting task is facilitated compared to the random condition.

Additionally, a stronger suppression of higher-alpha and beta power at posterior electrodes was observed for anticipated interruptions shortly before and after the onset of the retro-cue (see Fig. 5C). The suppression of posterior alpha power has been linked to the focusing of attention on the level of working memory^24,49^. Importantly, it was shown that this process can also be initiated proactively when expecting the time at which certain working memory content is required for report^50^. These oscillatory effects can therefore be related to a facilitated re-focusing of attention on the working memory task in expectation of the presentation of the retro-cue when the interruption has been anticipated.

Trials without interruptions, on the other hand, showed an opposite effect on oscillatory power in the alpha (see Fig. 5D) and beta frequency range before and during the presentation of the retro-cue (see Fig. 5E). There was more suppression of oscillatory power for the random than for the ‘no-interruption anticipated’ condition, with strongest effects at right-hemispheric parieto-occipital electrodes and left-hemispheric central electrodes (see Fig. 5G). With respect to the above-mentioned relationship between the suppression of oscillatory power in the alpha/beta frequency range and attentional orienting on the level of working memory, this might indicate that in the random condition attention had to be re-focused on the primary task before retro-cue presentation even without an interrupting task. This would mean that even in the absence of an interruption, the possibility of being interrupted may to some degree have distracted attention from the working memory task. Furthermore, even in the pre-stimulus interval (i.e., before presentation of the memory array), posterior alpha power was reduced more strongly when it was possible to anticipate the absence of an interruption (see Fig. 5F). This might indicate that participants could focus on the processing of the primary task better during blocks of trials without interruptions. Summarized, this pattern of results suggests that the benefit of anticipating the interruption condition was associated with the facilitation of the processing of the interruption task and resumption of the primary task after an interruption. In trials without an interruption, the modulations in the alpha and beta frequency range imply that the mere possibility of being interrupted drew attentional resources from the processing of the working memory task.

Contrary to our expectations, no significant differences between the age groups were found regarding the effects of anticipation on EEG level. Behavioral studies on task switching showed that older as well as younger participants benefit from anticipation of an upcoming task switch in the form of a reduction of switching costs^18^. Age-related differences in the reduction of switching costs through anticipation emerged mainly for tasks with high working memory load^51^. In fact, in the current study, only the older group showed a behavioral advantage in the primary and secondary tasks resulting from the anticipation of the interruption condition. However, this difference was not reflected in the EEG measures. In this regard, it should be noted that the observed effects of anticipation on neural oscillatory patterns were generally rather small and possible differences between the age groups may have been too weak to show up in our sample. Furthermore, it is possible that differences between the age groups in dealing with the anticipation of the interruption conditions, which were evident at the behavioral level, are rather reflected in EEG parameters which were not the focus of the present investigation (e.g., ERP effects related to the allocation of mental resources).

The present results can also be interpreted in terms of the concept of "dual mechanism of cognitive control", according to which cognitive processing can be more proactive or reactive in nature^52^. In proactive control, cognitive resources are provided in advance to ensure performance in an upcoming interruption. Regarding the processing of interruptions during a working memory task, proactive control should preferentially be applied when sufficient resources are available, when the interruption is reliably announced, and when it is cognitively demanding. In contrast, reactive control should be less associated with preparation for the interruption and thereby lead to poorer performance as a function of the cognitive demands of the interrupting task. Thus, the shift from proactive to reactive control assumed during aging^52^ might contribute to a deterioration in the handling of interruptions based on a less efficient use of possibilities to prepare for task interruptions with increasing age. Previously, it was shown that only younger but not older adults use proactive control when an interruption is announced at short notice during the trial^3^. However, the current results suggest that it is well possible to change processing strategies in anticipation of an interruption even with higher age, if one can adapt to the experimental condition across a set of multiple trials. This argues against a general age-related deficit regarding proactive cognitive control mechanisms but suggests that older adults need more preparation time to use proactive control mechanisms in an efficient way.

## Conclusion

In summary, it can be concluded that, on the one hand, older participants suffered more from being interrupted, which could be due to a higher difficulty focusing attention back on the memory representations required for completing the primary task. On the other hand, older adults could use the information about the upcoming experimental condition to adjust to the processing of the working memory task with or without interruption. However, this strategy was not sufficient to fully compensate for age-related deficits in the handling of interruptions. Younger subjects showed a generally better performance in dealing with interruptions but did not benefit in their behavioral performance from anticipating the experimental conditions. One possible reason for this could be that they still had sufficient working memory resources to process both tasks simultaneously, even in the condition with random interruptions. This could be investigated in future studies by independently manipulating the difficulty of both tasks within the experimental design.

## Methods

### Participants

A total of 38 young (age range: 18–30) and 28 older (age range: 55–70) right-handed participants attended the experiment. Three of the younger participants had to be excluded from the main experiment since they did not perform above chance level in the secondary (mathematical) task after three training sessions. Additionally, data from 2 young participants were not usable due to technical issues. One of them could not finish the experiment since the hardware crashed midway, and the event latencies from the EEG triggers were completely missing from the other participant. Finally, data of 1 young and 3 older participants were classified as outliers and were excluded from analysis (see below). This resulted in 32 young (*M*_*age*_ ± *SD* = 23.19 ± 3.56, 21 females, 11 males) and 25 older (*M*_*age*_ ± *SD* = 63.56 ± 4.31, 13 females, 12 males) participants being included in the final analysis. This sample size was determined by the fact that we wanted to have at least 24 valid datasets per age group, based on an earlier experiment on age differences in the processing of interruptions^15^. Due to problems in recruiting older participants, we decided to use free appointments for testing younger participants, thus leading to the higher number of datasets in the younger group. All participants were tested for right-handedness with an adapted questionnaire based on the Edinburgh Handedness Inventory. None of them reported any known neurological or psychiatric disorders. Before the experiment began, each participant was tested for normal color vision by means of the Ishihara Test for Color Blindness. They were compensated for participation with 12 € per hour or an equivalent amount of course credits and gave written informed consent. The study was approved by the ethics committee of the Leibniz Research Centre for Working Environment and Human Factors, and it was conducted in accordance with the Declaration of Helsinki.

### Experimental procedure

Before starting the experiment, each participant was asked to complete a set of neuropsychological tests which included the Trail-Making-Test (TMT Part A & B), Color-Word-Test and Number Repetition Forwards & Backwards. There was no statistically significant difference between younger and older adults regarding the number repetition task which measures verbal working memory performance and is included in the ‘Nürnberger Altersinventar’^53^. However, the dealing with interference as measured in the NAI version of the color-word-test was affected in older compared to younger adults and both information processing speed and selective attention (measured in the TMT Part A and B) were found to be worse for the older group (see Table 1). Importantly, no participant showed consistently deviant scores in these tests relative to the age-specific norm, suggesting that there was no special case of age-related cognitive impairment. As further support for this notion, the older participants completed the Mini-Mental-Status-Test prior to the main experiment and all of them passed this test with a score above 27, which rules out the prevalence of dementia within the older group.

**Table 1:** The full demographics of the sample and the results from neuropsychological tests for young and older participants.

	Young Adults (N = 32)	Older Adults (N = 25)	
Sex (female/male)	21/11	13/12	
Age (years)	23.19 ± 3.56, (18–30)	63.56 ± 4.31, (55–69)	
TMT Part A	21.92 ± 5.35, (11.30–37.31)	30.72 ± 10.34, (16.05–51.39)	t(33.96) = −3.87,
			*p* < .001
TMT Part B	47.23 ± 13.37, (21.53–90.62)	71.03 ± 24.38, (37.31–143.43)	t(35.1) = −4.39,
			*p* < .001
Color-word-test	7.75 ± 3.46, (2.63–17.06)	14.17 ± 7.11, (5.2–37.27)	t(32.82) = −4.15,
			*p* < .001
Number repetition task	12.19 ± 1.55, (9–15)	12.32 ± 2.27, (9–17)	t(40.61) = −0.25,
			*p* = .8

The experiment took place in a dimly lit chamber. Participants were sitting in front of a 21-inch CRT monitor (viewing distance: 110 cm; refresh rate: 100 Hz; resolution: 2048*1536). The task was programmed and run using Lazarus IDE (Free Pascal), and the stimuli were presented via ViSaGe MKII Stimulus Generator (Cambridge Research Systems, Rochester, UK). Each trial began with the presentation of a memory array on a grey background (background color in CIE1931 space: 0.287, 0.312, 15), which was composed of two randomly oriented blue bars (CIE1931: 0.195, 0.233, 42; size: 1° × 0.1°) presented at the center of their respective quadrants of the screen, always lateral to the center of the screen, and two randomly oriented orange bars on the opposite side (CIE1931: 0.484, 0.451, 42; size: 1° × 0.1°). The color presented on the left vs. right side of central fixation was randomly selected for each trial. Participants were informed that they had to focus either on the blue or orange bars for the entirety of the experiment and remember their orientation throughout each trial. The target color was counterbalanced across participants. The memory array was presented for 200 ms and followed by a central fixation cross (CIE1931: 0.287, 0.312, 40; length of each bar: 0.95° × 0.95°), which was either presented without interruption until the presentation of the retrospective cue (retro-cue) or replaced by a centrally presented summation task in the trials with interruption. In this summation task, participants had to indicate by pressing the left or right computer mouse button whether the equation (digit height: 0.95°) was solved correctly or incorrectly (which was the case in 50% of the trials). The mapping of ‘correct’ vs. ‘incorrect’ responses to the computer mouse buttons was counterbalanced across participants. This interruption task was presented for 2500 ms, during which the participants had to respond.

After the presentation of the interruption task (or the prolonged fixation phase), there was an interval of 800 ms with a fixation cross before the retro-cue was presented for 200 ms (total height: 1.23°), indicating whether the upper vs. lower bar from the memory array would be relevant for later orientation report. Following an additional 800 ms of fixation cross presentation, a black bar (CIE1931: 0.287, 0.312, 0; size: 1° × 0.1°) in random orientation was presented as the memory probe. Participants had to move the computer mouse on the horizontal axis to rotate the bar to match its orientation to the target bar and had to click the left computer mouse button to finalize their answer. This bar was either presented for a maximum of 4000 ms or, if the participant responded quicker, was replaced with a fixation cross for the duration of the inter-trial interval. The inter-trial interval varied between 500 and 1000 ms, with the jittering interval being drawn from a uniform distribution.

Before every set of ten trials (i.e., a sub-block), an announcement was made on the display to inform that in the following ten trials, the participant would be either always interrupted (interruption anticipated), never interrupted (no-interruption anticipated), or might be interrupted on a random basis (interruption vs. no-interruption random). During each block, participants were presented with two ‘interruption anticipated’ and two ‘no-interruption anticipated’ sub-blocks, and four random sub-blocks. Thus, the number of trials added up to 80 per block and the conditions were balanced over the course of the whole experiment. For each block, the order of these sub-blocks was shuffled. In the end, there were 120 trials per condition (‘interruption anticipated’, ‘no-interruption anticipated’, ‘interruption random’, ‘no-interruption random’), leading to a total of 480 trials. With all preparations considered, the experiment took around 3 h. Before the experiment began, the participants attended a training session at least once to introduce them to the task. The training session consisted of 30 trials, 10 trials from each sub-block type (interruption, no-interruption, random interruption vs. no-interruption). They were set to continue with the main experiment when they were able to perform more than 50% on the interrupting task (above chance level). If this criterion was not met, participants were asked to repeat the training session for a maximum of two more times.

### Behavioral data

Trials in which participants did not respond to the primary task (and to the interrupting task if it was an interruption condition) were considered incomplete and excluded from further analyses. Furthermore, trials in which the response to either task was faster than 150 ms (referring to the time of the computer mouse button press in the primary working memory task and in the interrupting task if it was an interruption condition) were excluded based on the assumption of premature responding. Two behavioral parameters were derived from the primary task: (a) The absolute difference between the actual orientation of the cued bar and the participants’ response in degrees (*the angular error*) and (b) the time it took them to start moving the mouse after memory probe onset as an equivalent of response times (*the response onset time*). The response onset time was chosen as a parameter, since prior investigations on the effect of task interruptions on working memory performance showed that it is influenced by the properties of the previous interrupting task (e.g., task difficulty^30^). Further behavioral parameters were derived from the interrupting task: (a) The response times, and (b) the accuracy, which was calculated as the ratio between the correct answers and the respective number of trials where the participant responded to the interrupting task. We further included an outlier detection procedure for primary task performance in the ‘no-interruption anticipated’ condition, which was assumed to be the cognitively least demanding condition. We excluded the data of participants who were detected as outliers in this experimental condition within their age group, determined by the interquartile range of the data. Participants’ data were discarded when the mean angular error (in degrees) was higher than the third quartile plus one and a half of the calculated interquartile range. This was specifically done to exclude participants who were either not able to focus on the task, not fully understood the task instructions or were not able to perform the task for other reasons. The same procedure was also applied to the response times of the interrupting task in the ‘interruption anticipated’ condition, which is supposed to be the least demanding interruption condition. This step was added to exclude those participants who did not perform the interrupting task as fast as possible. For the behavioral parameters in the primary task, mixed-design ANOVAs were used with Interruption (*interruption* vs. *no-interruption*), Anticipation (*interruption anticipated* vs. *random*), and Age (*young* vs. *older*) as independent factors. For the behavioral parameters in the interrupting task, a mixed-design ANOVA was run to check for effects of Anticipation (*interruption anticipated vs. random*) and Age (*young vs. older*). If further post-hoc t-tests were implemented, Bonferroni-Holm correction was used to account for multiple testing.

### Electrophysiological data

The electrophysiological data were recorded with a sampling rate of 1 kHz using a 64-channel passive-electrode cap (Easycap 64 Ch-Braincap, Easycap GmbH, Herrsching, Germany) with an extended 10/20 scalp configuration. FCz was used as the reference during recording and AFz as the ground electrode. All electrode impedances were kept below 10 kΩ. The signals were amplified for recording via a NeurOne Tesla TMS Amplifier (Bittium Bio- signals Ltd, Kuopio, Finland).

The collected data was then processed by EEGLAB v2022.1^54^, running on MATLAB v9.14.0 (R2023a, Mathworks, Natick, USA). First, EEG data was high-pass and then low-pass filtered with cut-off frequencies of 1 and 45 Hz (pop_eegfiltnew, FIR, Hamming windowed sinc, high-pass filter order = 3300, low-pass filter order = 330). Following that, flat line channels (clean_flatlines) and noisy channels (pop_rejchan) were rejected using a kurtosis criterion (SD = 5). On average, 3.7 channels were rejected (SD = 2.1, range: 0–7). The data was then re-referenced to average, and missing channels were interpolated (pop_interp, spherical).

To clear out artefacts arising from eye blinks or external noise, Independent Component Analysis^55^ was used. A copy of the original data was reorganized into 8 s epochs relative to memory array onset (−1000 to 7000 ms). Artefact trials were rejected via an automated trial rejection process (pop_autorej, threshold: 1000 µV, probability threshold: 5 SD, maximum rejection of trials per iteration: 5%). Then the data were downsampled to 250 Hz and ICA was run (infomax, estimating the number of sub-Gaussian sources, PCA to control for the reduced rank resulting from average reference and excluded channels). Resulting components were automatically classified via the ICLabel algorithm^56^ and components with a probability higher than 50% to stem from eye movements, eye blinks, line noise, or channel noise was rejected. On average, 4.9 components were rejected (SD = 3.0, range: 1–13). The calculated component weights and ICLabel results were transferred to the original dataset. These original data were then again segmented into epochs of 8 s relative to memory array onset (-1000 to 7000 ms). Finally, trials with large voltage fluctuations were rejected (pop_eegthresh, threshold low: -150 µV, threshold high: 150 µV), which resulted in an average of 25 trials rejected from the original data (SD = 35.1).

### Oscillatory power

Oscillatory power was calculated using wavelet convolution per channel with a Full-Width-at-Half-Maximum range of 750 to 100 ms, and extracting frequencies in logarithmic steps in the range of 4 to 30 Hz. An interval before memory array onset (-500 to -200 ms) was used as the condition-general oscillatory baseline. This means that we used a singular baseline across all conditions to be able to explore condition-specific processes relative to this baseline throughout the whole epoch (including the pre-stimulus interval). This resulted in time–frequency decompositions of the trials from 700 ms before to 6685 ms after the memory array onset, which were then down-sampled to 30 Hz to ease the calculation time of the following analyses. For further statistical analyses, an implementation of cluster-based permutation statistics by FieldTrip^57^ was run on the time–frequency data. The data were organized as subject*channel*frequency*time, first treating the sample as a whole and not separating young and older participants into two different groups (ft_freqstatistics, estimation method: Monte-Carlo, correction method: cluster, alpha level for a cluster to be significant: 0.05, parameter to collect from the clusters: maximum of the t-statistic sum, minimum number of channels for a cluster to be defined: 2, two-sided t-tests, alpha to control for the false alarm rate: 0.05, number of randomizations: 1000). The neighbor relations of channels were defined by the distance to each other and was prepared using an automated process (ft_prepare_neighbours). The clusters were selected as significant if their p-values exceeded 0.05, which was calculated by finding the percentile where the t-sum of the said cluster was positioned on the aggregated maximum of t-sum values over permutations (or more simply, by counting how many measurements out of permutations was equal to or greater than the t-sum of the cluster and dividing it by the total number of permutations). Since each significant cluster resulted in a large amount of significant time–frequency points for several channels, it was difficult to plot reasonable topographies for the observed effects. Therefore, we provide a metric indicating how strongly each channel contributed to the overall cluster by calculating the ratio between the significant datapoints per channel and the number of datapoints for the channel contributing the largest time–frequency cluster. Channels with values equal to or higher than 0.7 were further used to visualize the effects on time–frequency contour plots (see Figs. 5 and 6). Additionally, since the interrupting task induced high magnitude spectral perturbations whereas prolonged fixation periods did not, the time after the presentation of the retro-cue (presented 5000 ms after the memory array) was used for the interruption and no-interruption comparison, ensuring that only the selected conditions differed, and remaining aspects were the same for the given time range.

After analyzing the effects of anticipation and interruption on electrophysiological measures, we ran further cluster-based permutation statistics with age as a between-subjects factor and either the anticipation or interruption conditions as within-subject factor. Instead of just comparing absolute measures of conditions (e.g., random vs anticipated interruptions), the effects were calculated and compared between age groups (i.e., random minus anticipated interruptions, young vs older participants). In case of significant clusters found this way, we ran further post-hoc statistical comparisons (i.e., when there was a significant cluster with an *age x interruption* interaction, for example, the effect of the additional factor anticipation on oscillatory power in this cluster was tested in an ANOVA).

### Software packages

In addition to mentioned software packages such as EEGLAB and FieldTrip, several others were used for different purposes, including NumPy 1.23.4^58^ and Pandas 1.5.1^59^ for handling the data and descriptive statistics, Pingouin 0.5.3^60^ for ANOVAs and post-hoc t-tests in Python 3.9.14, with Matplotlib 3.5.3^61^ and seaborn 0.12.0^62^ for data visualization.

### Supplementary Information

Supplementary Information.

## Data Availability

Data and code are publicly available on the Open Science Framework (OSF) platform from the time of publication: (a public link will be added).
