# Supplementary material for: Be prepared for interruptions: EEG correlates of anticipation when dealing with task interruptions and the role of aging
Source: Sci Rep. 2024 Mar 7;14:5679. doi: 10.1038/s41598-024-56400-y (PMC10920752; doi:10.1038/s41598-024-56400-y)
Supplement: Supplementary file 1 — Supplementary Information. [file 41598_2024_56400_MOESM1_ESM.docx]

## Supplementary materials

1. **Effects of task interruptions on neural oscillations across all datasets (i.e., independent from age group)**


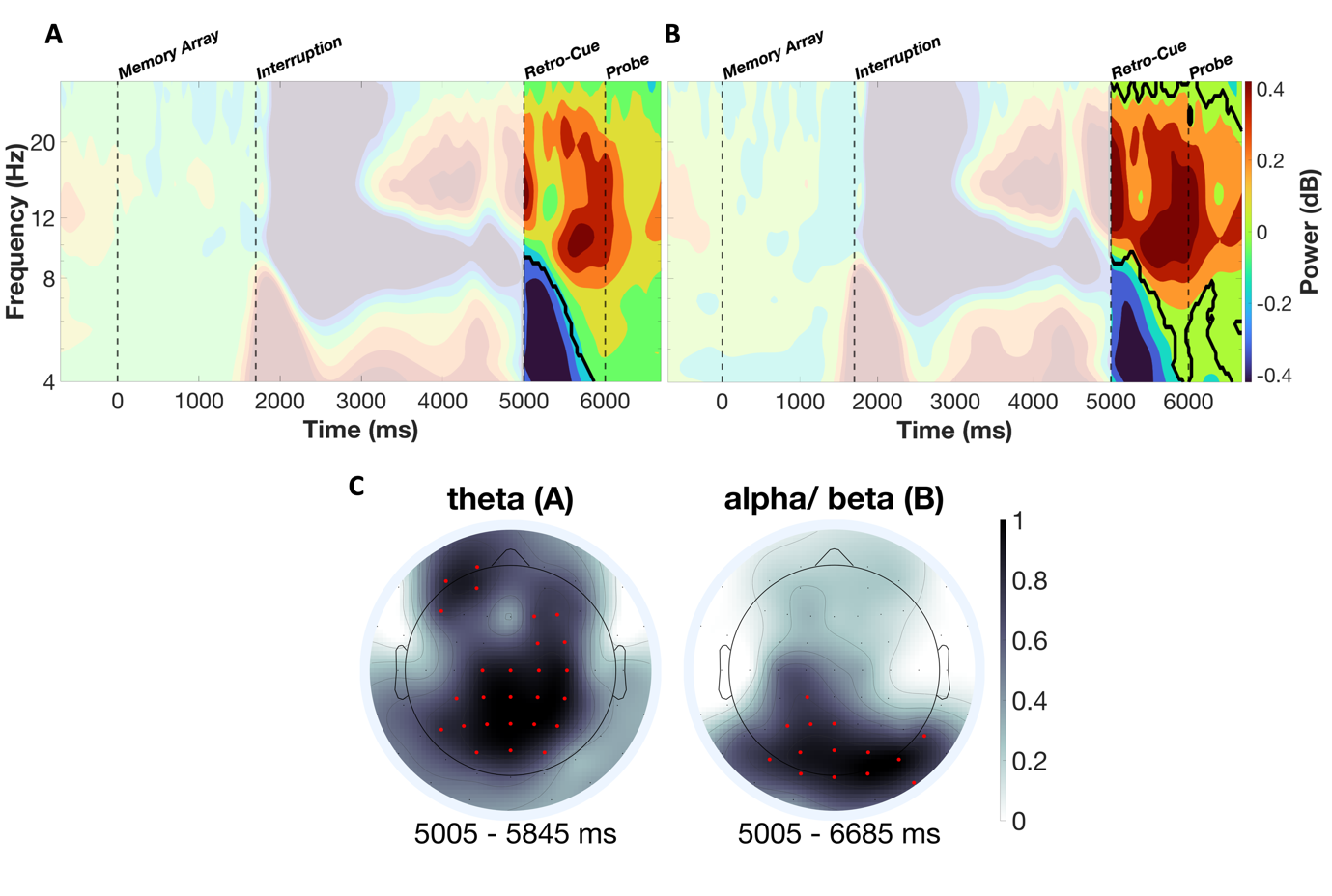


***Figure S1.*** *Time-frequency plots for the effects of interruptions, using the whole sample as one group. The two subplots (A and B) are for the difference between interruption and no-interruptions (averaged over anticipation condition). Note that for the analysis only the time following the retro-cue was considered for statistical testing. The events (onsets of memory array, interruption, retro-cue, and probe) are marked by vertical dotted lines. The topographies (C) are for each cluster per comparison, showing the localization of the effects. The amount of significant time-frequency points is normalized by the maximum of given cluster to obtain a contribution scale for each channel. The channels that exceeded the given threshold are marked in red, which were also used for the plots above.*

1. **Posterior alpha power asymmetries**

The bar stimuli in the memory array were presented in a lateralized way, meaning that the two relevant bars were presented either on the left or the right side of central fixation. This experimental design allowed for specifically calculating the contralateral (the mean of targets left, right-sided channels and target right, left-sided channels) vs. ipsilateral portions (the mean of targets left, left-sided channels and target right, right-sided channels) of the EEG signal. This way, we analyzed the asymmetry of alpha power (8 – 14 Hz) per experimental condition (interruption random vs. anticipated, no-interruption random vs. anticipated) separately for each age group. The parameters used for the wavelets were identical to those described in the methods section, except for the fact that no oscillatory baseline was used (which is required for the way the lateralization was calculated, see below). Channel pairs P5-P6, P7-P8, PO3-PO4 and PO7-PO8 were used for calculating the alpha power asymmetries over lateral parietal and parieto-occipital areas. The results, with average lateralized alpha power in a frequency range of 8-14 Hz, are depicted in figure S2.

There was a stronger suppression of oscillatory power contralateral than ipsilateral to the side of the task-relevant information right after memory array onset. However, when running a cluster-based permutation procedure (see methods section) on the time points between memory array and memory probe onset including all datasets (i.e., not separated by age groups), we did not find any statistically significant clusters for the comparison of the interruption vs. no-interruption conditions. There was also no significant cluster when comparing the anticipated vs. random conditions (see figure S2).

***
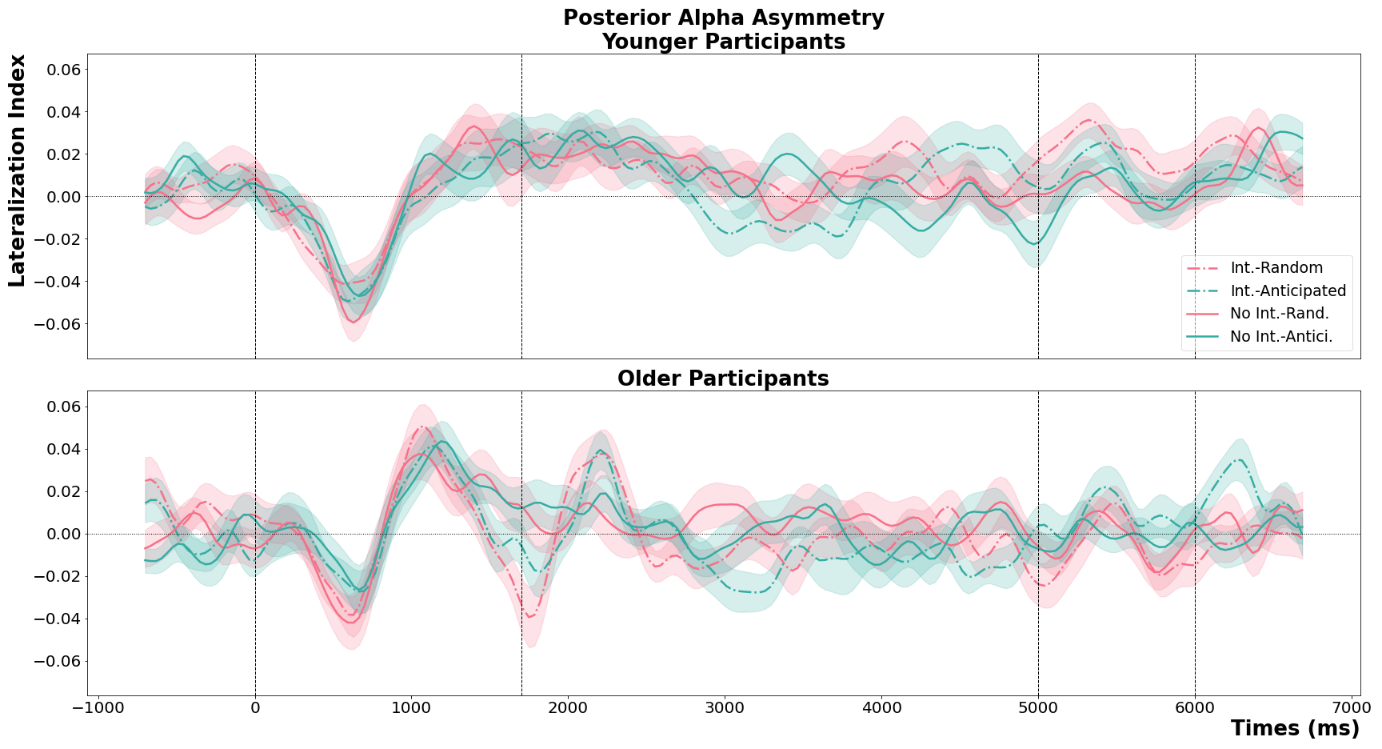
***

***Figure S2.*** *Posterior alpha power asymmetry (8-14 Hz average) separated for age groups and experimental conditions. The Lateralization Index refers to the contralateral minus ipsilateral difference in oscillatory power, divided by the sum of contralateral and ipsilateral oscillatory power (µV^2^/Hz). Negative values indicate the orienting of attention towards the location of the task-relevant information from the memory array.*

1. **Relation between neural oscillations after retro-cue presentation and primary task performance**

To investigate how the oscillatory power in the theta frequency range and the suppression of alpha and beta power after the retro-cue, which differed significantly between trials with and without interruption, are linked to performance in the working memory task, we used a median-split approach based on performance in the primary task. In all experimental conditions, we calculated the median performance (angular error and response onset times). The trials were then categorized into high vs. low performance trials for each experimental condition. Based on the significant time-frequency clusters when comparing interruption and no-interruption conditions (see figure S1), we ran mixed ANOVAs with the within-subject factors ‘interruption’ (interruption vs. no-interruption), ‘anticipation’ (anticipated vs. random) and the between-subjects factor ‘age’. In these ANOVA, we included a further within-subject factor for performance (high vs. low performance trials). These analyses were conducted separately for the theta and alpha-beta time frequency clusters and the two parameters for primary task performance (angular error and response onset times). Due to the exploratory nature of these analyses, P-values from the ANOVA were corrected for multiple testing by means of the Bonferroni-Holm procedure (p_adj_).

*
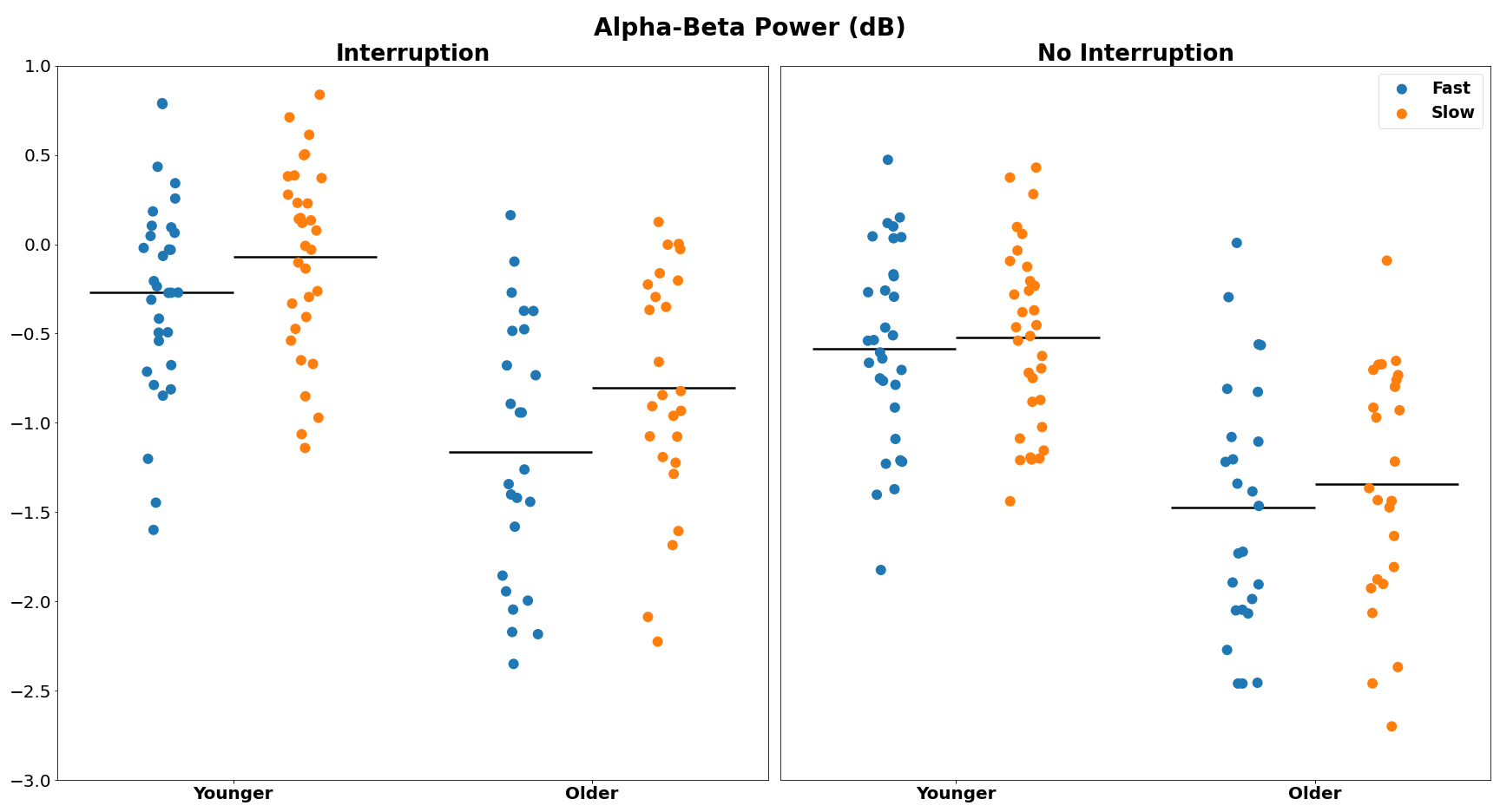
*

***Figure S3.*** *Scatterplots of alpha-beta oscillatory power after the retro-cue for both the younger and older age group, separated by trials with fast vs. slow response onset times.*

While for theta power there were no significant effects related to primary task performance (p_adj_ > 0.15), the suppression of alpha and beta power after retro-cue presentation differed as a function of both working memory accuracy and response onset times: There was a stronger suppression of alpha and beta power after the retro-cue in trials with high accuracy, F(1,55)=9.202, p_adj_=0.048. Furthermore, there was a performance main effect, F(1,55)=27.154, p_adj_<0.001, and an interruption * performance interaction when trials were split based on the median of response onset times, F(1,55)=17.239, p_adj_<0.001. As shown in figure S3, these results indicate that faster response onset times followed stronger alpha-beta suppression after the presentation of the retro-cue, particularly in trials including an interruption.
